# ﻿A review of the *Strongylophthalmyiacoarctata* subgroup (Diptera, Brachycera, Strongylophthalmyiidae) from China, with the descriptions of three new species

**DOI:** 10.3897/zookeys.1168.104699

**Published:** 2023-07-04

**Authors:** Jiale Zhou, Neal L. Evenhuis, Ding Yang

**Affiliations:** 1 Department of Entomology, College of Plant Protection, China Agricultural University, 2 Yuanmingyuan West Road, 100193 Beijing, China China Agricultural University Beijing China; 2 Department of Natural Sciences, Bernice Pauahi Bishop Museum, 1525 Bernice Street, Honolulu, 96817-2704 Hawaii, USA Department of Natural Sciences, Bernice Pauahi Bishop Museum Honolulu United States of America

**Keywords:** Acalyptratae, Diopsoidea, East Asia, Oriental Realm, Palearctic Realm, taxonomy

## Abstract

The species belonging to the *Strongylophthalmyiacoarctata* subgroup of the *S.punctata* group (Diptera: Brachycera: Strongylophthalmyiidae) from China are reviewed. Six species are recognized, including three new species: *S.corniculata***sp. nov.**, *S.flagellicornis***sp. nov.**, and *S.tangwangana***sp. nov.***Strongylophthalmyianarwhal* Evenhuis, 2020 and *S.raricornis* Shatalkin, 1981 are recorded from China for the first time, and *S.raricornis* is also recorded from South Korea for the first time. An identification key to the Asian species of the *S.coarctata* subgroup is provided.

## ﻿Introduction

The acalyptrate family Strongylophthalmyiidae currently contains 90 species divided into two genera: the monotypic *Nartshukia* Shatalkin, 1993 from Vietnam, and the speciose *Strongylophthalmyia* Heller, 1902 from the Australasian, Nearctic, Oriental, and Palearctic realms (Evenhuis 2016, 2020; Galinskaya and Shatalkin 2018). Members of *Strongylophthalmyia* are mostly assigned into four species-groups (*S.crinita* group, *S.fascipennis* group, *S.punctata* group, and *S.ustulata* group), with nearly 40% of species belonging to the *S.punctata* group, while the other 12 species are unplaced and two are treated as nomen dubia (Shatalkin 1996; Evenhuis 2016). The *S.punctata* group is characterized by the highly modified male antennal first flagellomere having a variable process, and is further divided into the *S.coarctata* subgroup (10 spp.) and *S.punctata* subgroup (25 spp.) (Shatalkin 1996; Evenhuis 2016).

The Chinese species belonging to the *S.coarctata* subgroup are reviewed in the present study. Prior to this study, only one species of this subgroup, *S.coarctata* Hendel, 1913, had been recorded from China (Yang and Wang 1998; Qilemoge et al. 2021). Here we describe three new species, and record two further species for the Chinese fauna and one new to the South Korean fauna. A key to the species of the *S.coarctata* subgroup from Asia is provided.

## ﻿Materials and methods

Specimens examined or cited in this study are deposited in the following institutions:

**NHMUK**Natural History Museum, London, UK;

**CAU**Entomological Museum of China Agricultural University, Beijing, China;

**CSCA**California State Collection of Arthropods, California Department of Food & Agriculture, Sacramento, USA;

**QSBG**Queen Sirikit Botanic Garden, Chiang Mai, Thailand;

**SDEI**Senckenberg Deutsches Entomologisches Institut, Müncheberg, Germany;

**ZMUM** Zoological Museum of Moscow State University, Moscow, Russia.

Male terminalia were prepared by macerating the apical portion of the abdomen in heated 10% KOH solution for approximately 10 min, and then rinsing in distilled water. External structure and terminalia were examined using a Nikon SMZ745 stereoscopic microscope. After examination, the terminalia were transferred to fresh glycerin and stored in microvials pinned below the corresponding specimens.

Photographs were taken using a Canon 7D Mark II digital camera with a Canon micro lens MP-E 65 mm for habitus, and an Olympus BX51 microscope for terminalia. Figures were stacked with Helicon Focus v. 5.3 and assembled by Adobe Photoshop 2020. The distribution map was prepared using the online version of SimpleMappr (Shorthouse 2010). Terminology follows Evenhuis (2016) and Lonsdale (2020); we use the term “phallic plate” following Lonsdale (2020) to refer the structure posteroventral to the basiphallus, although the homology of this structure is arguable. Measurements were obtained using a calibrated micrometer. Distributional data are given from specimens examined in this study and from literature records; new distributional records are marked by asterisk (*); data from literature records (without specimen examination) are given the source.

## ﻿Taxonomy

### ﻿Genus Strongylophthalmyia

Taxon classificationAnimaliaDipteraStrongylophthalmyiidae

Heller, 1902

5761FCDF-85CB-557D-8F93-0F52DB564E11

Strongylophthalmus Hendel, 1902: 179. Type species: Chylizaustulata Zetterstedt, 1847, by original designation. Preoccupied by Strongylophthalmus Mannheim, 1853 (Insecta: Coleoptera).
Strongylophthalmyia Heller, 1902: 226. Replacement name for Strongylophthalmus Hendel, 1902.
Labropsila Meijere, 1914: 24. Type species: Labropsilapolita Meijere, 1914, by subsequent designation (Hennig 1941: 36). Synonymized by Meijere (1916: 87).

#### Notes.

For an extensive treatment of the family, systematic considerations, definitions, a key to species-groups, and a checklist of global species, see Evenhuis (2016) and Lonsdale (2020).

##### ﻿The *Strongylophthalmyiacoarctata* subgroup

**Diagnosis.** Recognized within the genus by the following characters: antennal first flagellomere of male modified, having variable antennal process; arista bare; fore femur of male simple, lacking thorn-like spicules dorsally.

**Diversity and distribution.** This subgroup currently contains ten described species, most of which are distributed in the Oriental Realm, while one species occurs in the northeastern Palearctic and three species are endemic to Papua New Guinea (Evenhuis 2016, 2020). *Strongylophthalmyiacoarctata* Hendel was previously known from China (Taiwan). Five species, including 3 described herein, are now added to the Chinese fauna.

### ﻿Key to species of *Strongylophthalmyiacoarctata* subgroup from Asia (based on males)

**Table:** 

1	Antennal first flagellomere bifid, with dorsal and ventral processes (Fig. 38)	**2**
–	Antennal first flagellomere with a single process below insertion of arista (Figs 1, 6, 17, 27, 49)	**3**
2	Antennal processes short and stout, distinctly shorter than arista (Fig. 38); mesonotum with 7 dorsocentral setae (Fig. 39); femora blackish brown (Fig. 34)	***Strongylophthalmyiararicornis* Shatalkin**
–	Antennal processes extremely long and thin, distinctly longer than arista; mesonotum with 1 dorsocentral seta; femora yellow, hind femur with a dark brown subapical ring	***Strongylophthalmyiamekistocera* Evenhuis**
3	Antennal process short, distinctly shorter than arista (Figs 6, 49)	**4**
–	Antennal process long, distinctly longer than arista (Figs 1, 17, 27)	**6**
4	Frons yellowish brown in anterior half (Fig. 4); antennal first flagellomere with a short, conical process (Fig. 6)	***Strongylophthalmyiacorniculata* sp. nov.**
–	Frons entirely black; first antennal flagellomere with a small, blunt process (Fig. 49)	**5**
5	Antennal first flagellomere yellowish brown with dorsal half dark brown; hind tibia darkened in middle	***Strongylophthalmyiagibbifera* Shatalkin**
–	Antennal first flagellomere yellowish brown (Fig. 49); hind tibia largely dark brown (Fig. 45)	***Strongylophthalmyiatangwangana* sp. nov.**
6	Antennal process slender, wipe-like, placed dorsally on first flagellomere in close proximity to arista (Figs 1, 17)	**7**
–	Antennal process thick, sword-like, placed anteriorly on first flagellomere (Fig. 27)	***Strongylophthalmyianarwhal* Evenhuis**
7	Frons partly to entirely yellow; antennal process covered with dense black setulae (Fig. 17); wing with dark suffusion at apex (Fig. 19)	**8**
–	Frons entirely black; antennal process covered with dense whitish setulae (Fig. 1); wing fully hyaline	**9**
8	Frons black with anterior part yellow; antennal first flagellomere bicolorous, ovate; wing with median transverse band at level of dm-m	***Strongylophthalmyiafreidbergi* Shatalkin**
–	Frons entirely yellow (Fig. 15); antennal first flagellomere yellow, subrhombic (Fig. 17); wing without median transverse band at level of dm-m (Fig. 19)	***Strongylophthalmyiaflagellicornis* sp. nov.**
9	Antennal first flagellomere ovate (Fig. 1); postpronotum and propleuron partly yellowish brown (Figs 1, 2)	***Strongylophthalmyiacoarctata* Hendel**
–	First antennal flagellomere subrhombic; thorax entirely black	***Strongylophthalmyiastylocera* Shatalkin**

### ﻿ Strongylophthalmyia coarctata

Taxon classificationAnimaliaDipteraStrongylophthalmyiidae

Hendel, 1913

6DF7E80D-1B03-5A6B-83D0-77E56495176D

Figs 1 , 2

Strongylophthalmyia coarctata Hendel, 1913: 87 (protologue); Hennig (1940: 309) (in key, figure); Steyskal (1971: 142) (in key); Rohlfien and Ewald (1972: 413) (type material, record); Steyskal (1977: 21) (catalogue, distribution); Yang and Wang (1998: 458) (in key, distribution, record); Lin and Chen (1999: 68) (listed); Papp (2005: 195) (record); Shatalkin (2007: 560) (type material, redescription); Iwasa and Evenhuis (2014: 103) (listed, distribution); Evenhuis (2016: 207) (listed, distribution); Qilemoge et al. (2021: 236) (catalogue, distribution). Syntypes (2♂4♀): China, Taiwan, Hengchun, SDEI.

#### Material examined.

China. Taiwan: Pingtung, Manzhou, Kankau [= Gangkou], 1912.iv, leg. H. Sauter (1♂, NHMUK).

#### Diagnosis.

**Figures 1, 2.**
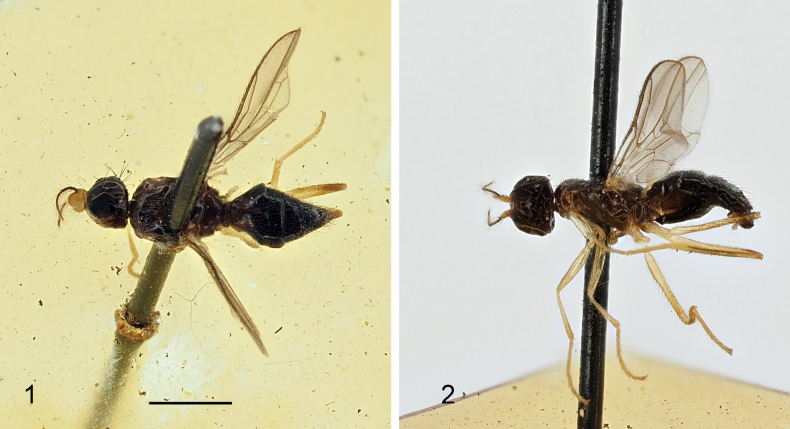
*Strongylophthalmyiacoarctata* Hendel, 1913, non-type male, habitus: **1** dorsal view **2** lateral view. Scale bar: 1 mm.

Generally shiny black (Figs 1, 2); antennal first flagellomere of male yellow, ovate (Fig. 1), with a long slender process covered with dense white setulae (Figs 1, 2); mesonotum blackish, postpronotum and propleuron partly yellowish brown (Figs 1, 2); wing hyaline (Fig. 2); mid and hind femora yellow, with apex dark brown (Figs 1, 2); distiphallus with small apical “glans” (see Hennig 1940: fig. 11).

#### Distribution.

China – Taiwan: Chiayi (Papp 2005), Kaohsiung (Rohlfien and Ewald 1972), Pingtung (Fig. 56).

#### Remarks.

This species was originally described based on “Mehrere ♀, 2♂” (syntype) from “Kankau (Koshun)” (now Hengchun, Pingtung, Taiwan, China) (Hendel 1913). According to Rohlfien and Ewald (1972), the type material of this species was preserved in the collection of SDEI and included two males and four females. Shatalkin (2007) redescribed this species based on a male and a female which he referred as “holotype” and “paratype”, respectively, and mentioned that these specimens were deposited in the Museum für Naturkunde, Berlin, Germany (ZMHB). However, no such specimens could be found in the collection of ZMHB (J. Pohl, pers. comm.), and the collection of SDEI was unavailable to us during the present study. We therefore presume that the type material of this species is still kept in SDEI, and Shatalkin (2007) made an incorrect interpretation on its depository. The uses of the term “holotype” and “paratype” by Shatalkin (2007) did not constitute valid lectotype designations (ICZN 1999, Art. 74.7), therefore the status of the type specimens of this species should remain as syntypes.

During the present study, we have examined a male from “Kankau” (now Gangkou, Pingtung, Taiwan, China) in the collection of NHMUK. Images of the dorsal and lateral habitus of this specimen (Figs 1, 2) are provided herein for facilitating the identification of this species.

### ﻿ Strongylophthalmyia corniculata sp. nov.

Taxon classificationAnimaliaDipteraStrongylophthalmyiidae

C098D7A0-A563-5507-BBAD-CAA9509ACE32

https://zoobank.org/13CA1D3F-B33D-4BEA-8B27-3EFEA6E19FC2

Figs 3 , 4–8 , 9–12

#### Type material.

***Holotype*** (♂): China, Yunnan, Honghe, Lvchun, Huanglianshan, 2018.v.19, by Malaise trap (CAU).

#### Diagnosis.

**Figure 3.**
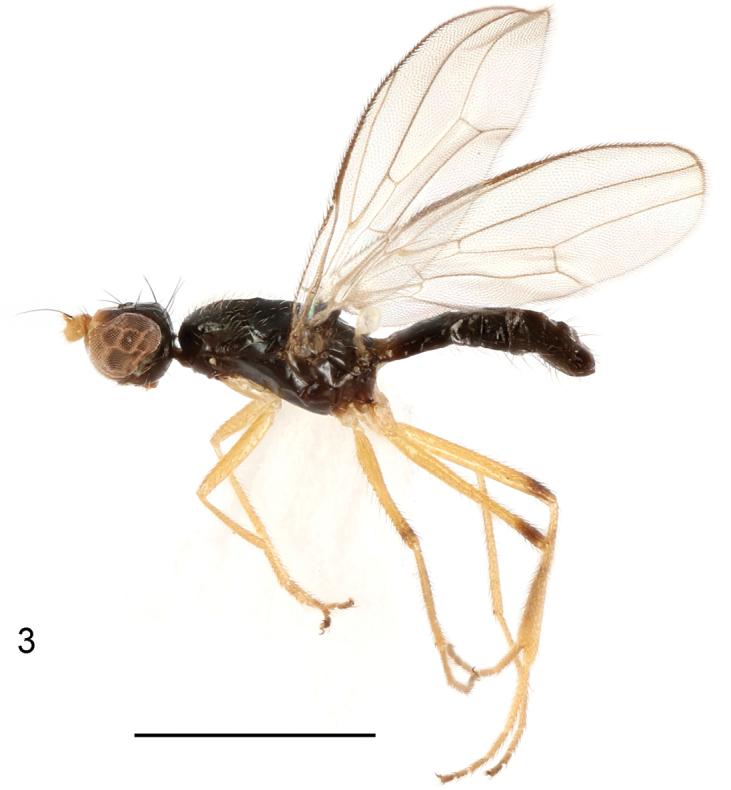
*Strongylophthalmyiacorniculata* sp. nov., male, holotype, habitus, lateral view. Scale bar: 1 mm.

**Figures 4–8.**
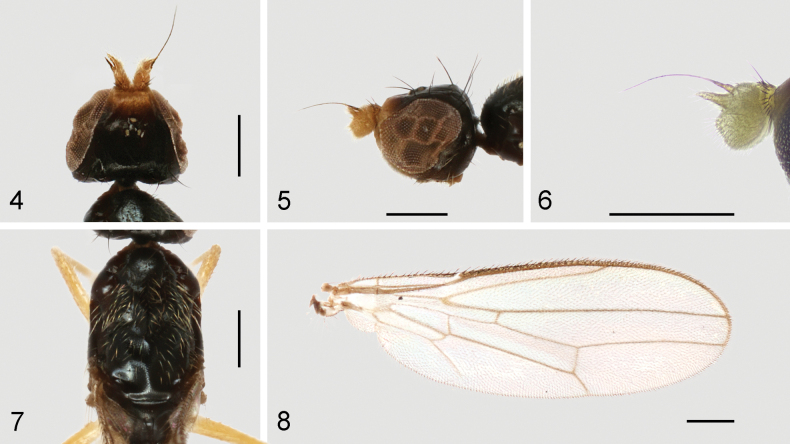
*Strongylophthalmyiacorniculata* sp. nov., male, holotype: **4** head, dorsal view **5** same, lateral view **6** left antenna, lateral view **7** thorax, dorsal view **8** wing. Scale bars: 0.25 mm.

Generally shiny black (Fig. 3); anterior half of frons yellowish brown (Fig. 4); antennal first flagellomere of male yellowish brown, ovate, with a short conical process (Figs 5, 6); wing hyaline (Fig. 8); mid and hind femora yellow, with narrow dark brown ring subapically (Fig. 3); hind femur of male lacking inner basal process; distiphallus less than half as long as phallapodeme, lacking apical “glans” (Fig. 12).

**Figures 9–12.**
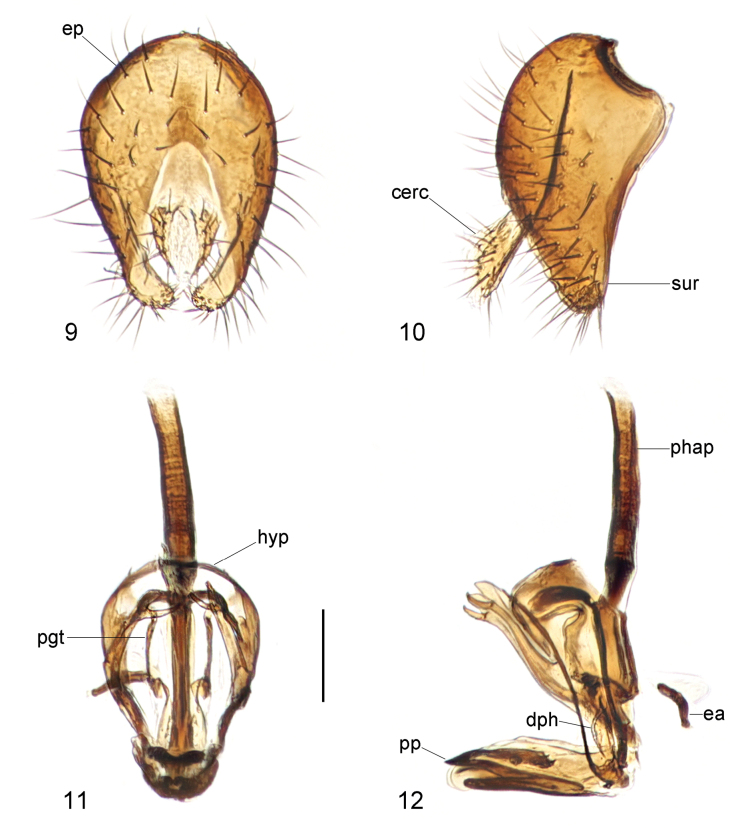
*Strongylophthalmyiacorniculata* sp. nov., male genitalia: **9** external genitalia, posterior view **10** same, lateral view **11** internal genitalia, ventral view **12** same, lateral view. Abbreviations: cerc = cercus; dph = distiphallus; ea = ejaculatory apodeme; ep = epandrium; hyp = hypandrium; pgt = pregonite; phap = phallapodeme; pp = phallic plate; sur = surstylus. Scale bar: 0.1 mm.

#### Description.

Body length 2.2 mm, wing length 2.2 mm.

**Male.** Generally shiny black (Fig. 3). Face, parafacial and anterior half of frons yellowish brown (Figs 4, 5). Antenna yellowish brown with arista dark brown (Fig. 5). Proboscis and palpus brown. Wing hyaline (Fig. 8); wing veins brown to dark brown. Halter white with base slightly darkened. Legs yellow with mid and hind femora narrowly dark brown subapically (Fig. 3); tarsomeres 4 and 5 dark brown.

Head (Figs 4, 5) with frons finely inflated; parafacial with dense silvery tomentose stripe; gena with silvery tomentose stripe along eye margin; postgena bulging. Head chaetotaxy: 1 inner vertical seta, 1 outer vertical seta, 3 fronto-orbital setae, 1 ocellar seta, 1 postocellar seta. Clypeus band-like; palpus elongate, with long sparse golden setulae. Antennal scape with scattered marginal setae and 1 dominant dorsal seta; pedicel with single strong seta dorsally; first flagellomere (Figs 5, 6) ovate, wider than long, densely covered with long white setulae, with a short conical process dorsally; antennal process (Fig. 6) with short dense white setulae, 0.7× as long as first flagellomere, sharp at apex; arista longer than antennal process.

Thorax with mesonotum (Fig. 7) densely covered with short scattered golden setulae, in dorsal view with distinct transverse suture. Anepisternum with short setulae along notopleural suture. Scutellum (Fig. 7) subtriangular, broad, slightly inflated. Thoracic chaetotaxy: 1 postpronotal seta, 1 anepisternal seta, 2 notopleural setae, 1 dorsocentral seta, 1 posterior supra-alar seta, 1 scutellar seta. Wing (Fig. 8) with R_4+5_ and M_1+2_ almost parallel apically; apical section of M_1+2_ finely arched; M_4_ and CuA+CuP not reaching but very closely approaching wing margin; r-m located near basal one-third (0.36) of cell dm; apical section of M_4_ nearly as long as dm-m; alula small; anal lobe slightly narrowed. Legs with dense whitish yellow setulae; hind femur lacking inner basal process.

Abdomen covered with long dense setae. Tergite 1 weakly sclerotized. Pregenital sclerites relatively weakly sclerotized.

Male genitalia: Epandrium (Figs 9, 10) short and broad, with long dense setae. Surstylus (Figs 9, 10) with short stout setae on inner distal surface. Cerci (Figs 9, 10) narrow, elongate, finger-like, with short dense setae. Hypandrium (Figs 11, 12) broadly rounded anteriorly, strongly arched medially, with one pair of bifid, long anterior lobes. Phallapodeme (Figs 11, 12) long, rod-like. Pregonite (Fig. 11) long, narrow, band-like, basally fused to inner surface of hypandrium. Phallic plate (Fig. 12) thickened, divided into two articulating sclerites. Distiphallus (Fig. 12) extremely short, less than half as long as phallapodeme, lacking apical “glans”, membrane microtrichose. Ejaculatory apodeme (Fig. 12) small, slightly curved.

**Female** unknown.

#### Etymology.

The specific epithet is derived from Latin *corniculata*, referring to the short conical antennal process of this new species.

#### Distribution.

China – Yunnan: Lvchun (Fig. 56).

#### Comparative notes.

This new species is similar to *S.gibbifera* Shatalkin, 1993 from Vietnam in that both have an ovate first flagellomere with a small, short antennal process, and a fully hyaline wing. The new species differs from *S.gibbifera* in the following characters: anterior half of frons yellowish brown (frons entirely black in *S.gibbifera*); first flagellomere yellowish brown (yellowish brown with dorsal half dark brown in *S.gibbifera*); antennal process conical, with sharp apex (short and blunt in *S.gibbifera*); mid and hind femora narrowly dark brown subapically, hind tibia largely darkened (femora yellow with hind femur weakly darkened at apex, hind tibia darkened in middle in *S.gibbifera*).

### ﻿ Strongylophthalmyia flagellicornis sp. nov.

Taxon classificationAnimaliaDipteraStrongylophthalmyiidae

82F2C50F-B92B-537E-95A7-52C08CCD0F4F

https://zoobank.org/AA20A164-85FA-4937-8975-DACDE96FBFC9

Figs 13–14 , 15–19 , 20–23

#### Type material.

***Holotype*** (♂): China, Yunnan, Xishuangbanna, Mengla, Yaoqu, 840 m, 2020.xi.22, leg. Liang Wang (CAU). ***Paratypes***: Same collection data as for holotype (9♂, CAU); China, Yunnan, Honghe, Gejiu, Lvshuihe, 505 m, 2019.iii.30, leg. Xin Li & Liang Wang (5♂1♀, CAU); China, Yunnan, Honghe, Lvchun, Huanglianshan, 1300 m, 2018.vi.16, leg. Liang Wang (2♂, CAU).

#### Diagnosis.

**Figures 13, 14.**
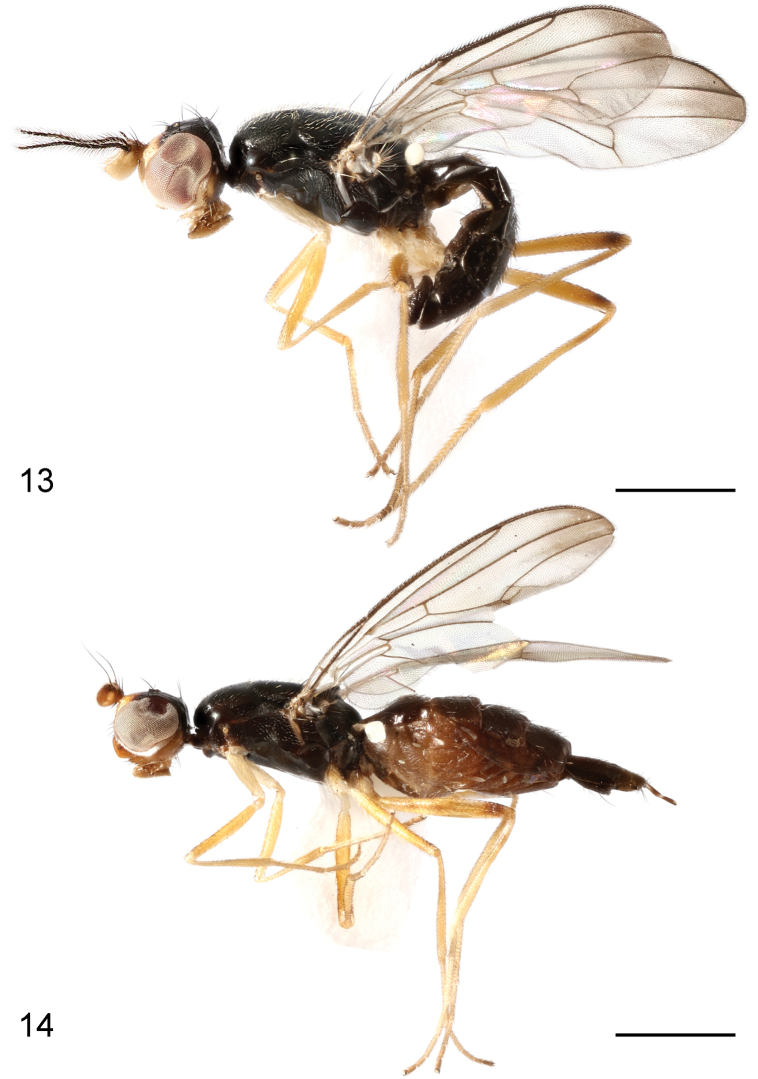
*Strongylophthalmyiaflagellicornis* sp. nov., lateral habitus, lateral view: **13** male, holotype **14** female, paratype. Scale bars: 1 mm.

**Figures 15–19.**
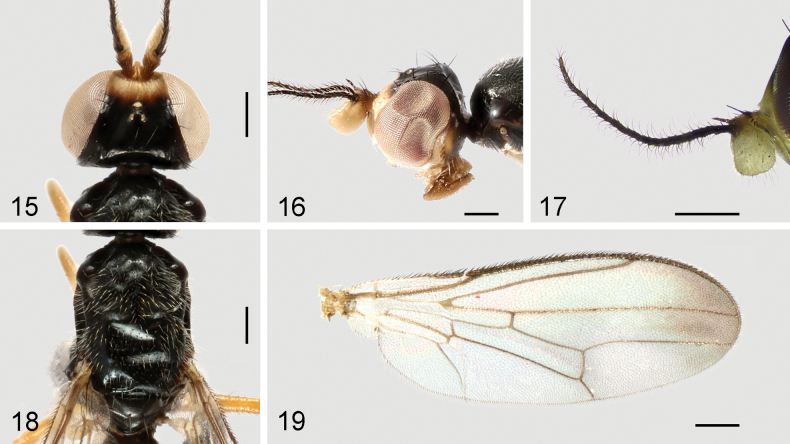
*Strongylophthalmyiaflagellicornis* sp. nov., male, holotype: **15** head, dorsal view **16** same, lateral view **17** left antenna, lateral view **18** thorax, dorsal view **19** wing. Scale bars: 0.25 mm.

Generally shiny black (Figs 13, 14); frons yellow (Fig. 15); antennal first flagellomere of male yellow, subrhombic, with a long slender process covered with dense black setulae (Figs 16, 17); wing infumate, with large dark suffusion at apex (Fig. 19); mid and hind femora yellow with narrow dark brown ring subapically (indistinct on mid femur) (Figs 13, 14); hind femur of male with one thorn-like inner basal process; distiphallus nearly as long as phallapodeme, with small apical “glans” (Figs 22, 23).

**Figures 20–23.**
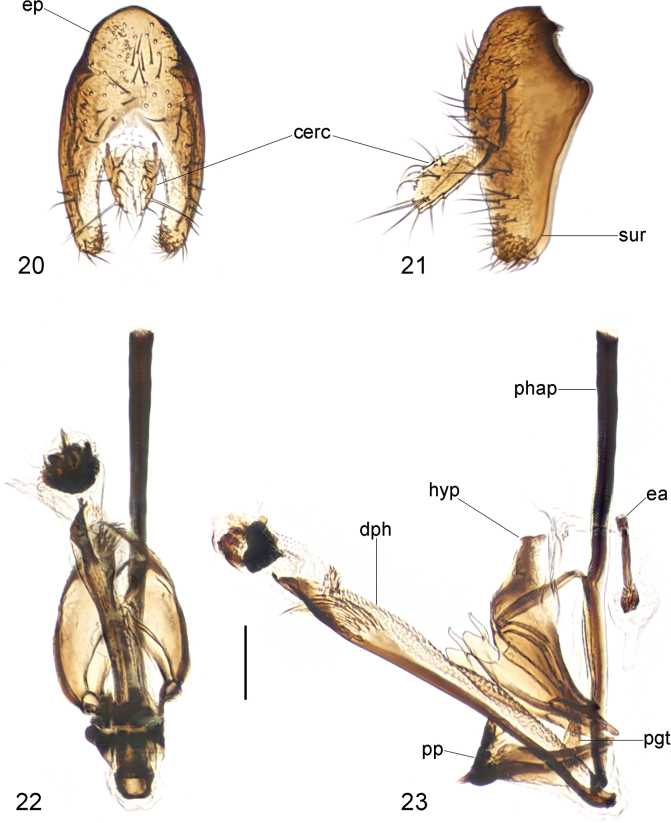
*Strongylophthalmyiaflagellicornis* sp. nov., male genitalia: **20** external genitalia, posterior view **21** same, lateral view **22** internal genitalia, ventral view **23** same, lateral view. Abbreviations: cerc = cercus; dph = distiphallus; ea = ejaculatory apodeme; ep = epandrium; hyp = hypandrium; pgt = pregonite; phap = phallapodeme; pp = phallic plate; sur = surstylus. Scale bar: 0.1 mm.

#### Description.

Body length 4.2–4.7 mm, wing length 2.4–3.0 mm.

**Male.** Generally shiny black (Fig. 13). Frons, face, parafacial, and gena yellow (Figs 15, 16). Antenna yellow; antennal process and arista blackish brown (Figs 16, 17). Clypeus yellow to yellowish brown; proboscis brown; palpus yellow. Wing infumate, with large dark suffusion at apex (Fig. 19); wing veins brown to dark brown. Halter white with base slightly darkened. Legs yellow; mid and hind femora with narrow dark brown ring subapically (indistinct on mid femur) (Fig. 13); mid and hind tarsomeres 4 and 5 dark brown. Abdominal syntergite 1+2 with large median yellow patch in posterior half.

Head (Figs 15, 16) with frons with minute shallow striations; parafacial with dense silvery tomentose stripe; gena with silvery tomentose stripe along eye margin; postgena bulging, with several white long setulae. Head chaetotaxy: 1 inner vertical seta, 1 outer vertical seta, 3 fronto-orbital setae, 1 ocellar seta, 1 postocellar seta. Clypeus band-like; palpus elongate, with short dense golden setulae. Antennal scape with scattered marginal setae and 1 dominant dorsal seta; pedicel with single strong seta dorsally; first flagellomere (Figs 16, 17) subrhombic, wider than long, densely covered with whitish yellow setulae, with a long slender process dorsally; antennal process (Fig. 17) with dense erect black setulae, 4.8× as long as first flagellomere; arista shorter than antennal process.

Thorax with mesonotum (Fig. 18) densely covered with short scattered golden setulae, in dorsal view with distinct transverse suture. Anepisternum with short dense golden setulae posteriorly. Scutellum (Fig. 18) subtriangular, broad, slightly inflated. Thoracic chaetotaxy: 1 anepisternal seta, 2 notopleural setae, 2 dorsocentral setae, 2 posterior supra-alar setae, 1 scutellar seta. Wing (Fig. 19) with R_4+5_ and M_1+2_ slightly convergent apically; apical section of M_1+2_ clearly arched; M_4_ and CuA+CuP not reaching but very closely approaching wing margin; r-m located at basal two-fifth (0.4) of cell dm; apical section of M_4_ shorter than dm-m; alula small; anal lobe well developed. Legs with dense dark setulae; hind femur with one thorn-like inner basal process.

Abdomen covered with short to long dense setae. Tergite 1 normally sclerotized. Pregenital sclerites weakly sclerotized.

Male genitalia: Epandrium (Figs 20, 21) long and narrow, subovate in lateral view, with long dense setae. Surstylus (Figs 20, 21) with short stout setae on inner distal surface. Cerci (Figs 20, 21) narrow, elongate, finger-like, fused along length, with 1 long subapical seta and several short setae. Hypandrium (Figs 22, 23) narrowly rounded anteriorly, arched medially, with one pair of bifid anterior lobes. Phallapodeme (Figs 22, 23) long, straight, rod-like, nearly as long as distiphallus. Pregonite (Fig. 22) very long, narrow, band-like, basally fused to inner surface of hypandrium. Phallic plate (Fig. 23) divided into two articulating sclerites. Distiphallus (Figs 22, 23) extremely long, nearly as long as phallapodeme, unsegmented, with sclerotized bands and apical “glans”, membrane microtrichose. Ejaculatory apodeme (Fig. 23) relatively long, straight, narrow.

**Female.** Antennal first flagellomere yellow with anterior margin darkened, ovate, lacking process (Fig. 14); clypeus thick, bulbous; hind femur lacking process; abdomen without yellow patch on syntergite 1+2. Other characters same as those of male.

#### Etymology.

The specific epithet is derived from Latin *flagell*- and -*cornis*, referring to the long, whip-like antennal process of this new species.

#### Distribution.

China – Yunnan: Gejiu, Lvchun, Mengla (Fig. 56).

#### Comparative notes.

This new species resembles *S.shatalkini* Iwasa & Evenhuis, 2014 from Papua New Guinea and *S.stylocera* Shatalkin, 1996 from Philippines by having the long and slender antennal process. The new species differs from *S.shatalkini* in the following characters: frons yellow (entirely black in *S.shatalkini*); antennal first flagellomere of male subrhombic, with antennal process 4.8× as long as first flagellomere (ovate, with antennal process 4× as long as first flagellomere in *S.shatalkini*); femora yellow with mid and hind femora narrowly dark brown subapically (femora uniformly dark brown in *S.shatalkini*); wing with distinct large dark suffusion at apex, and R_4+5_ and M_1+2_ slightly convergent apically (wing with broad median transverse band and faint small apical suffusion, and R_4+5_ and M_1+2_ almost parallel in *S.shatalkini*). The new species can be separated from *S.stylocera* in the following characters: frons yellow (entirely black in *S.stylocera*); antennal process of male with dense dark setulae (with white setulae in *S.stylocera*); wing with distinct large dark suffusion at apex (wing hyaline in *S.stylocera*); abdomen with a large median yellow patch on syntergite 1+2 in male (uniformly black in *S.stylocera*).

### ﻿ Strongylophthalmyia narwhal

Taxon classificationAnimaliaDipteraStrongylophthalmyiidae

Evenhuis, 2020

71E0657F-C315-5D50-9586-6048468ED52A

Figs 24 , 25–29 , 30–33

Strongylophthalmyia narwhal Evenhuis, 2020: 5 (protologue). Holotype (♂): Thailand, Chiang Mai, Chiang Mai, QSBG.

#### Type material examined.

***Holotype*** (♂): Thailand, Chiang Mai, Queen Sirikit Botanical Garden, 2013.vii.11–25, leg. M. Hauser, by Malaise trap (QSBG).

#### Other materials examined.

China. Yunnan: Honghe, Lvchun, Huanglianshan, 380 m, 2018.iv.15, leg. Liang Wang (1♂, CAU); same locality and collector as preceding, 1300 m, 2018.iv.16 (1♂, CAU); Xishuangbanna, Jinghong, Naban River Watershed National Nature Reserve, Mandian Waterfall, 700 m, 2020.xi.25, leg. Liang Wang (1♂, CAU).

#### Diagnosis.

**Figure 24.**
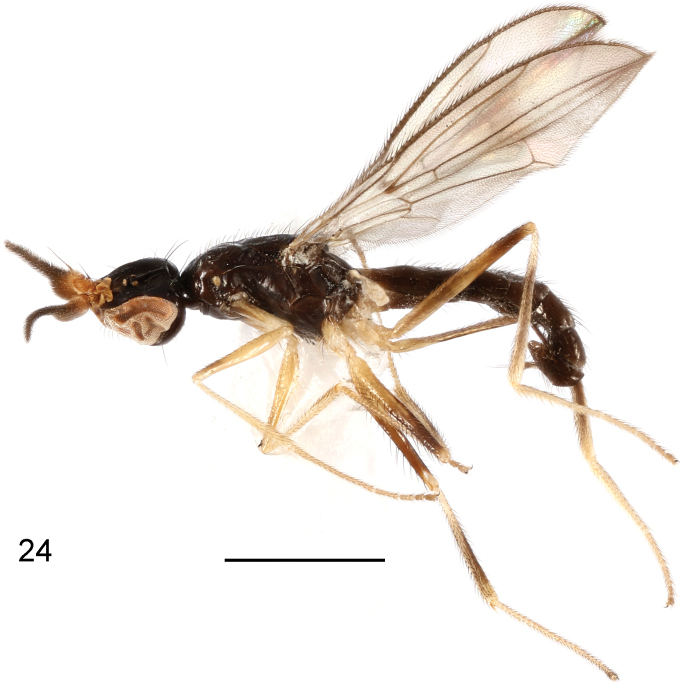
*Strongylophthalmyianarwhal* Evenhuis, 2020, non-type male, habitus, lateral view. Scale bar: 1 mm.

Generally shiny blackish brown (Fig. 24); antennal first flagellomere of male yellowish brown with apical half darkened, round, with a long, thick, sword-like process (Figs 26, 27); wing infumate, with large dark suffusion at apex and narrow median transverse band at level of dm-m (Fig. 29); fore femur yellow with narrow brown ring subapically (Fig. 24); mid and hind femora yellowish brown, with apical half of mid femur and broad subbasal and subapical rings of hind femur dark brown (Fig. 24); hind femur of male with one wart-like inner basal process; distiphallus approx. 1.3× as long as phallapodeme, with small apical “glans” (Figs 32, 33).

**Figures 25–29.**
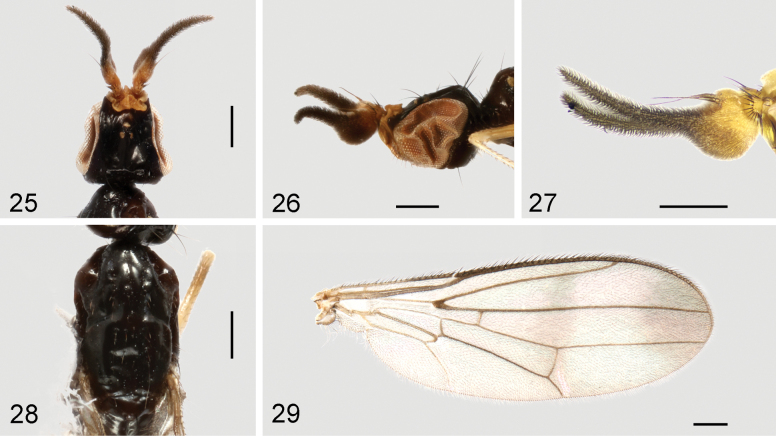
*Strongylophthalmyianarwhal* Evenhuis, 2020, non-type male: **25** head, dorsal view **26** same, lateral view **27** left antenna, lateral view **28** thorax, dorsal view **29** wing. Scale bars: 0.25 mm.

**Figures 30–33.**
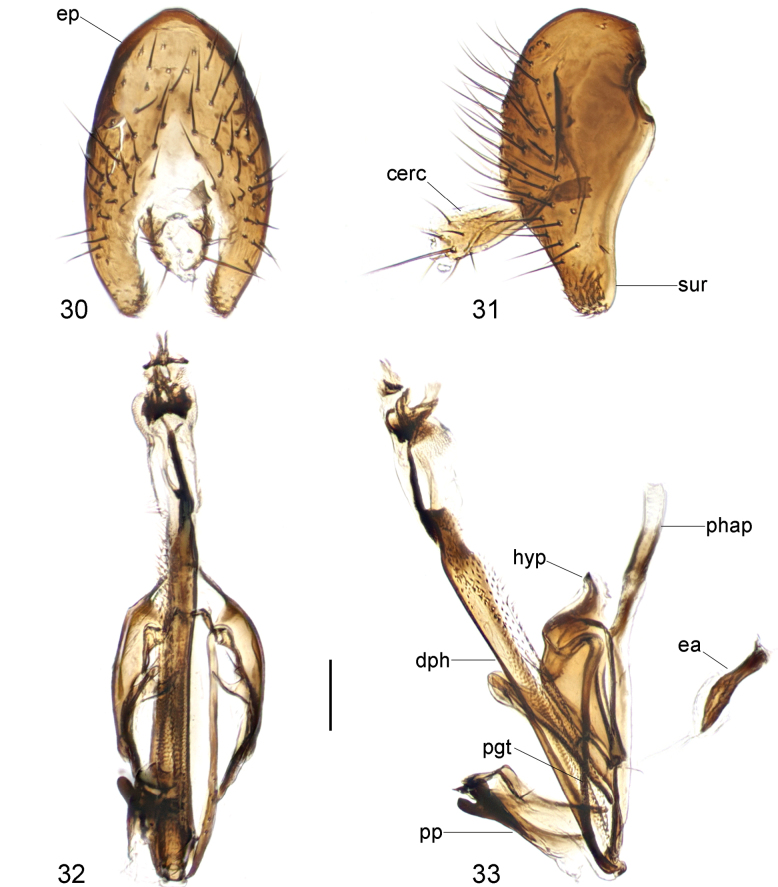
*Strongylophthalmyianarwhal* Evenhuis, 2020, male genitalia: **30** external genitalia, posterior view **31** same, lateral view **32** internal genitalia, ventral view **33** same, lateral view. Abbreviations: cerc = cercus; dph = distiphallus; ea = ejaculatory apodeme; ep = epandrium; hyp = hypandrium; pgt = pregonite; phap = phallapodeme; pp = phallic plate; sur = surstylus. Scale bar: 0.1 mm.

#### Description of male genitalia.

Epandrium (Figs 30, 31) short and narrow, with long, dense setae. Surstylus (Figs 30, 31) with short stout setae on inner distal surface. Cerci (Figs 30, 31) narrow, elongate, finger-like, fused along length, with 1 long subapical seta and several short setae. Hypandrium (Figs 32, 33) narrowly rounded anteriorly, strongly arched medially, with one pair of bifid anterior lobes. Phallapodeme (Figs 32, 33) short, slightly curved, distinctly shorter than distiphallus. Pregonite (Fig. 32) very long, narrow, band-like, basally fused to inner surface of hypandrium. Phallic plate (Fig. 33) divided into two articulating sclerites. Distiphallus (Figs 32, 33) extremely long, nearly 1.3× as long as phallapodeme, unsegmented, with sclerotized bands and apical “glans”, membrane microtrichose. Ejaculatory apodeme (Fig. 33) relatively large, slightly curved.

#### Distribution.

China – Yunnan: Jinghong*, Lvchun* (Fig. 56). Thailand – Chiang Mai: Chiang Mai (Evenhuis 2020).

#### Remarks.

This species was described based on a male holotype from Chiang Mai, Thailand (Evenhuis 2020). Newly available specimens from Yunnan, China are identical in most of the features described by Evenhuis (2020), but differs in the following aspects: (i) gena blackish brown as general body color; (ii) both wings with narrow median transverse band at level of dm-m; (iii) legs yellow to yellowish brown, with basal half of fore coxa, narrow subapical ring of fore femur, apical half of mid femur (except extreme apex), broad subbasal and subapical rings of hind femur, mid and hind tibiae (except bases and apices) and tarsomeres 4 and 5 brown to dark brown.

The chaetotaxy, which was not mentioned in the original description, can now be added as: head with 1 inner vertical seta, 1 outer vertical seta, 3 fronto-orbital setae, 1 ocellar seta and 1 postocellar seta; thorax with 1 anepisternal seta, 1 postpronotal seta, 2 notopleural setae, 2 dorsocentral setae, 2 posterior supra-alar setae and 1 scutellar seta; mid femur with row of long erect black setae. The male genitalia are described as above.

### ﻿ Strongylophthalmyia raricornis

Taxon classificationAnimaliaDipteraStrongylophthalmyiidae

Shatalkin, 1981

10F6A737-379E-5C14-896F-7EF57A263340

Figs 34–35 , 36–40 , 41–44

Strongylophthalmyia raricornis Shatalkin, 1981: 792 (protologue); Krivosheina (1984: 27) (catalogue, distribution); Shatalkin (1993: 124, 126) (in key, bionomics, figure); Shatalkin (1996: 156) (diagnosis, redescription, record, figure); Krivosheina (1999: 508) (in key, distribution, figure); Ozerov (2010: 156) (type material); Iwasa and Evenhuis (2014: 103) (listed, distribution); Evenhuis (2016: 207) (listed, distribution, figure). Holotype (♂): Russia, Khabarovsk Krai, Maly Khingan, ZMUM.

#### Material examined.

China. Beijing: Haidian, Jiufeng, 101 m, 2018.vi.20, leg. Jiale Zhou & Yike Cao (2♂1♀, CAU). Shaanxi: Mei County, Haopingsi, 1120 m, 2020.vi.29, leg. Bing Zhang (4♂, CAU); Tongchuan, Miaowan, 1233 m, 2019.vii.28, leg. Qicheng Yang & Jiaojie Wang (1♂10♀, CAU); Tongchuan, Yuhuagong, 1385 m, 2019.vii.30, leg. Jiaojie Wang (3♂, CAU); Tongchuan, Liulin, 1020 m, 2019.vii.27, leg. Qicheng Yang & Weijian Huang (4♂2♀, CAU); Xunyi, Shimenshan, 1577 m, 2019.vii.26, leg. Qicheng Yang (4♂, CAU); same locality, 1605m, 2019.vii.25, leg. Jiaojie Wang (1♂1♀, CAU). Shanghai: Baoshan, Gucun Park, 2021.v.9, photographed by Deyao Zhou (1♂, photo voucher only). Tianjin: Jizhou, Baxianshan, 221–706 m, 2019.vi.28, leg. Ding Yang (1♂4♀, CAU). SOUTH KOREA. Chungbuk: Okcheon, Dongi, Soesan, 150 m, leg. P. Tripton, by Malaise trap (1♂1♀, CSCA).

#### Diagnosis.

**Figures 34, 35.**
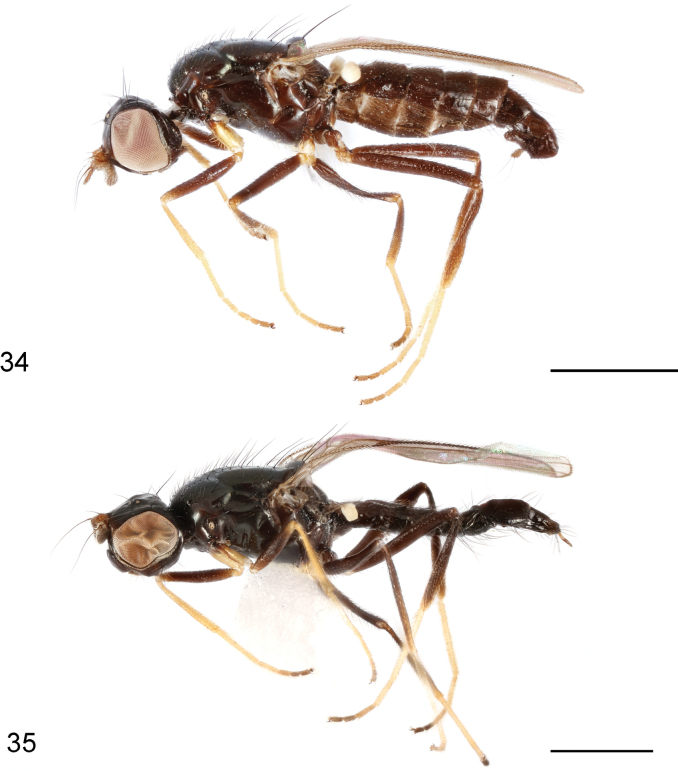
*Strongylophthalmyiararicornis* Shatalkin, 1981, habitus, lateral view: **34** non-type male **35** non-type female. Scale bars: 1 mm.

Generally shiny black (Figs 34, 35); antennal first flagellomere of male yellowish brown, bifid, C-shaped (Figs 37, 38); wing hyaline (Fig. 40); legs blackish brown with fore and mid coxae (except base), trochanters, extreme apex of fore and mid femora, fore tibia, apex of mid and hind tibiae, and tarsomeres 1–3 yellow (Figs 34, 35); hind trochanter of male with a tiny thorn-like process on internal surface; hind femur of male with three small warty inner basal processes, middle one bearing setae, other two bare; distiphallus slightly shorter than phallapodeme, with large apical “glans” (Figs 43, 44).

**Figures 36–40.**
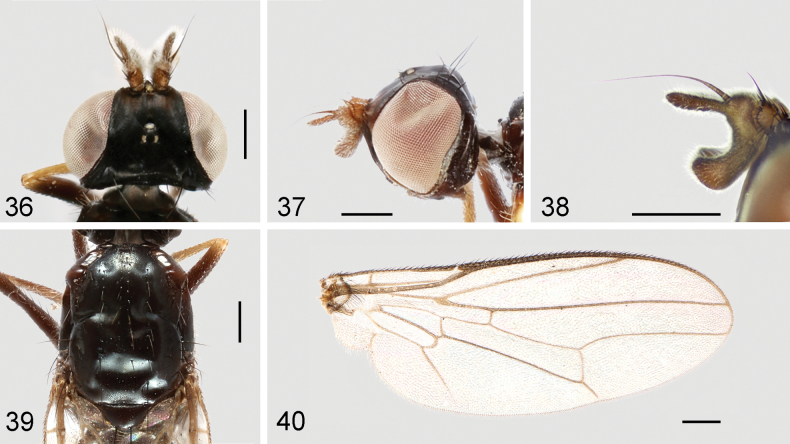
*Strongylophthalmyiararicornis* Shatalkin, 1981, non-type male: **36** head, dorsal view **37** same, lateral view **38** left antenna, lateral view; 39 thorax, dorsal view **40** wing. Scale bars: 0.25 mm.

**Figure 41–44.**
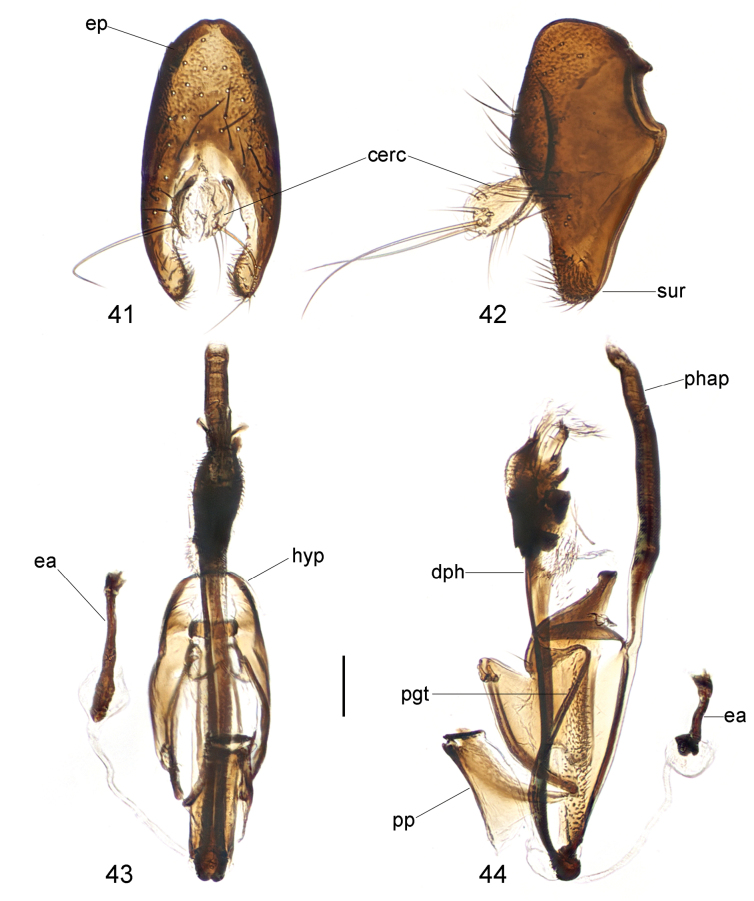
*Strongylophthalmyiararicornis* Shatalkin, 1981, male genitalia: **41** external genitalia, posterior view **42** same, lateral view **43** internal genitalia, ventral view **44** same, lateral view. Abbreviations: cerc = cercus; dph = distiphallus; ea = ejaculatory apodeme; ep = epandrium; hyp = hypandrium; pgt = pregonite; phap = phallapodeme; pp = phallic plate; sur = surstylus. Scale bar: 0.1 mm.

#### Redescription.

Body length 3.5–4.6 mm, wing length 2.5–2.9 mm.

**Male.** Generally shiny black (Fig. 34). Antenna yellowish brown with arista dark brown (Figs 37, 38). Proboscis and palpus brown. Wing hyaline (Fig. 40); wing veins brown to dark brown. Halter white with base slightly darkened. Legs blackish brown; fore and mid coxae (except base), trochanters, extreme apex of fore and mid femora, fore tibia, apex of mid and hind tibiae, and tarsomeres 1–3 yellow (Fig. 34).

Head (Figs 36, 37) with frons flattened; parafacial with dense silvery tomentose stripes; gena with silvery tomentose stripe along eye margin; postgena bulging. Head chaetotaxy: 1 inner vertical seta, 1 outer vertical seta, 3 fronto-orbital setae, 1 ocellar seta, 1 postocellar seta. Clypeus subquadrate; palpus elongate, with short dense black setulae. Antennal scape with scattered marginal setae and 1 dominant dorsal seta; pedicel with elongate dorsal seta; first flagellomere (Figs 37, 38) bifid, C-shaped, with short dense white setulae; arista longer than first flagellomere.

Thorax with mesonotum (Fig. 39) densely covered with long setulae located in rows, in dorsal view with distinct transverse suture. Anepisternum with short dense setulae. Katepisternum with short setulae ventrolaterally near mid coxa. Scutellum (Fig. 39) subtriangular, slightly inflated. Thoracic chaetotaxy: 1 anepisternal seta, 1 presutural intra-alar seta, 2 notopleural setae, 7 dorsocentral setae, 2 posterior supra-alar setae, 1 scutellar seta. Wing (Fig. 40) with R_4+5_ and M_1+2_ almost parallel apically; apical section of M_1+2_ clearly arched; M_4_ and CuA+CuP not reaching but very closely approaching wing margin; r-m located near basal one-third (0.34) of cell dm; apical section of M_4_ shorter than dm-m; alula relatively large; anal lobe well developed. Legs with dense dark setulae; fore coxa with several long white setulae antero-apically; hind trochanter with a tiny thorn-like process on internal surface; hind femur with three small warty inner basal processes, middle one bearing setae, other two bare.

Abdomen covered with short to long dense setae. Tergite 1 weakly sclerotized. Pregenital sclerites relatively weakly sclerotized.

Male genitalia: Epandrium (Figs 41, 42) long and narrow, subovate in lateral view, with long dense setae. Surstylus (Figs 41, 42) with several long dense setae at apex and short stout setae on inner distal surface. Cerci (Figs 41, 42) relatively short and broad, fused along length, with one rather long subapical seta and several short setae. Hypandrium (Figs 43, 44) narrowly rounded anteriorly, with one pair of bifid, long anterior lobes. Phallapodeme (Figs 43, 44) long, curved, slightly longer than distiphallus. Pregonite (Fig. 43) very long, narrow, band-like, basally fused to inner surface of hypandrium. Phallic plate (Fig. 44) divided into two articulating sclerites. Distiphallus (Figs 43, 44) extremely long, slightly shorter than phallapodeme, unsegmented, with sclerotized bands and large apical “glans”, membrane microtrichose. Ejaculatory apodeme (Figs 43, 44) large, slightly swollen at apex.

**Female.** Antennal first flagellomere ovate (Fig. 35); clypeus thick, bulbous; hind trochanter and femur lacking process. Other characters same as those of male.

#### Distribution.

China – Beijing: Haidian*, Shaanxi: Mei County*, Tongchuan*, Xunyi*, Shanghai: Baoshan*, Tianjin: Jizhou* (Fig. 56). Russia – Khabarovsk Krai: Maly Khingan (Shatalkin 1981), Primorsky Krai: Kamenushka, Lazovskiy Nature Reserve (Shatalkin 1996). South Korea – Chungbuk: Okcheon* (Fig. 56).

#### Remarks.

The male genitalia of this species were illustrated by Shatalkin (1996) and Krivosheina (1999) and are described here for the first time. This species was previously reported from the Russian Far East (Shatalkin 1996; Krivosheina 1999). Considering the new distributional records from China and South Korea, this species seems to be widely distributed in the eastern Palaearctic Realm.

### ﻿ Strongylophthalmyia tangwangana sp. nov.

Taxon classificationAnimaliaDipteraStrongylophthalmyiidae

A557E278-EF99-58B3-9A69-78950F26369D

https://zoobank.org/67840AD5-61F8-43CC-8724-03B5AF060A5F

Figs 45–46 , 47–51 , 52–55

#### Type material.

***Holotype*** (♂): China, Shaanxi, Tongchuan, Tang Wang Hunting Ground, 1429 m, 2019.vii.30, leg. Qicheng Yang (CAU). ***Paratypes*.** Same collection data as for holotype (1♂2♀, CAU).

#### Diagnosis.

**Figures 45, 46.**
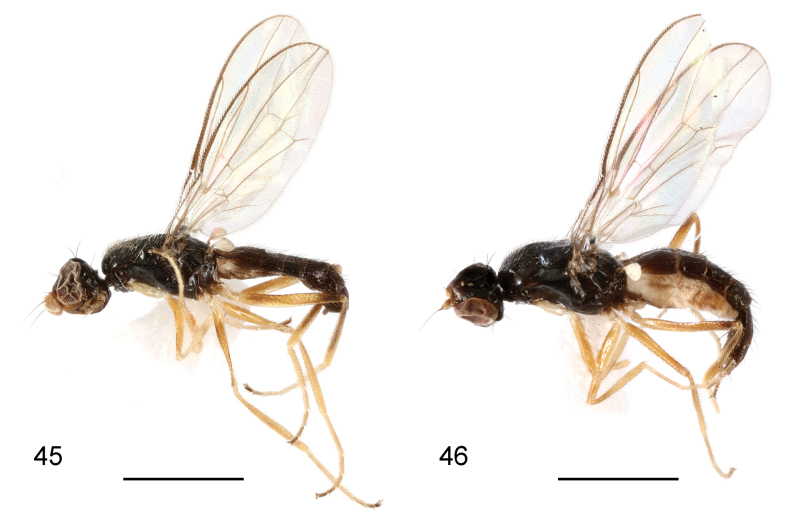
*Strongylophthalmyiatangwangana* sp. nov., habitus, lateral view: **45** male, holotype **46** female, paratype. Scale bars: 1 mm.

Generally shiny black (Figs 45, 46); antennal first flagellomere of male yellowish brown, ovate, with a small, bump-like process (Figs 48, 49); wing hyaline (Fig. 51); mid and hind femora yellow, with narrow dark brown ring subapically (indistinct on mid femur) (Figs 45, 46); hind femur of male lacking inner basal process; distiphallus nearly half as long as phallapodeme, lacking apical “glans” (Figs 54, 55).

**Figures 47–51.**
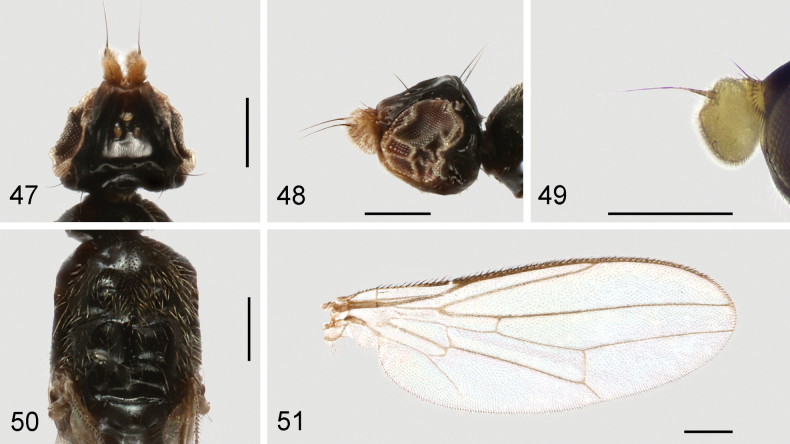
*Strongylophthalmyiatangwangana* sp. nov., male, holotype: **47** head, dorsal view **48** same, lateral view **49** left antenna, lateral view **50** thorax, dorsal view **51** wing. Scale bars: 0.25 mm.

**Figures 52–55.**
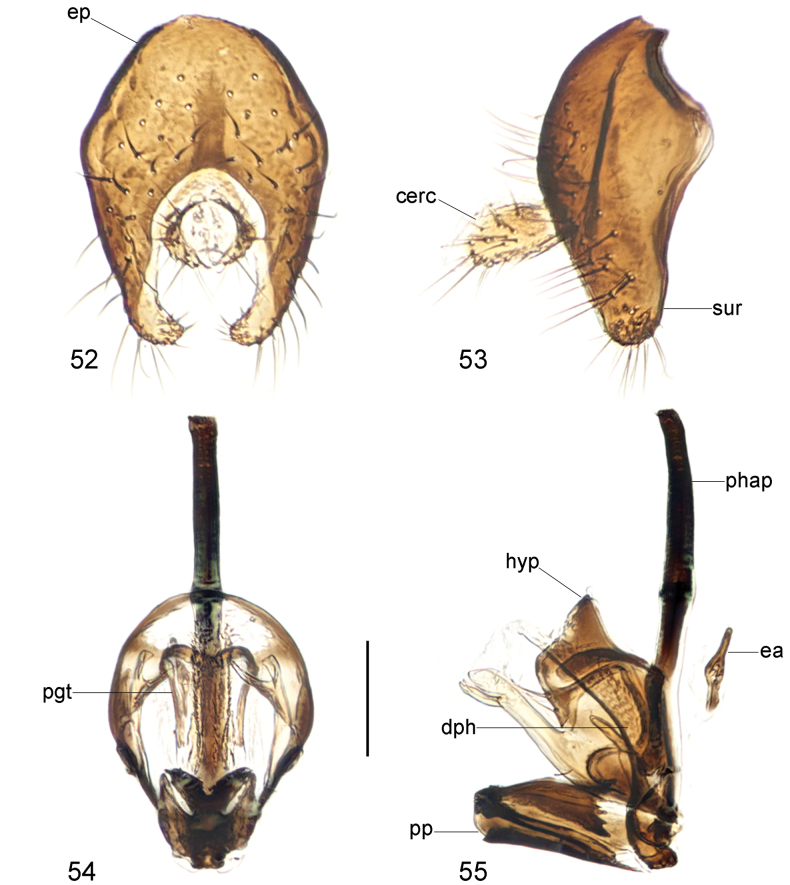
*Strongylophthalmyiatangwangana* sp. nov., male genitalia: **52** external genitalia, posterior view **53** same, lateral view **54** internal genitalia, ventral view **55** same, lateral view. Abbreviations: cerc = cercus; dph = distiphallus; ea = ejaculatory apodeme; ep = epandrium; hyp = hypandrium; pgt = pregonite; phap = phallapodeme; pp = phallic plate; sur = surstylus. Scale bar: 0.1 mm.

#### Description.

Body length 2.5–3.2 mm, wing length 2.1–2.4 mm.

**Male.** Generally shiny black (Fig. 45). Anterior margin of frons slightly paler; face and parafacial yellowish brown. Antenna yellowish brown with arista dark brown (Figs 48, 49). Proboscis and palpus yellowish brown. Wing hyaline (Fig. 51); wing veins brown to dark brown. Halter white with base darkened. Legs yellow; mid and hind femora with narrow dark brown ring subapically (indistinct on mid femur) (Figs 45, 46); hind tibia largely dark brown (Fig. 45); tarsomeres 4 and 5 dark brown.

Head (Figs 47, 48) with frons finely inflated; parafacial with dense silvery tomentose stripe; gena with silvery tomentose stripe along eye margin; postgena bulging. Head chaetotaxy: 1 inner vertical seta, 1 outer vertical seta, 3 fronto-orbital setae, 1 ocellar seta, 1 postocellar seta. Clypeus subquadrate; palpus elongate, with short dense golden setulae. Antennal scape with scattered marginal setae and 1 dominant dorsal seta; pedicel with single strong seta dorsally; first flagellomere ovate, wider than long, densely covered with short white setulae, with a small, bump-like process dorsally (Figs 48, 49); arista longer than first flagellomere.

Thorax with mesonotum (Fig. 50) densely covered with short scattered golden setulae, in dorsal view with distinct transverse suture. Anepisternum with short setulae posteriorly. Scutellum (Fig. 50) subtriangular, broad, slightly inflated. Thoracic chaetotaxy: 1 anepisternal seta, 2 notopleural setae, 1 dorsocentral seta, 2 posterior supra-alar setae, 1 scutellar seta. Wing (Fig. 51) with R_4+5_ and M_1+2_ slightly convergent apically; apical section of M_1+2_ straight; M_4_ and CuA+CuP not reaching but very closely approaching wing margin; r-m located near basal one-third (0.34) of cell dm; apical section of M_4_ shorter than dm-m; alula small; anal lobe slightly narrowed. Legs with dense whitish yellow setulae; fore coxa with several long white setulae antero-apically; hind femur lacking inner basal process.

Abdomen covered with long dense setae. Tergite 1 weakly sclerotized. Pregenital sclerites normally sclerotized.

Male genitalia: Epandrium (Figs 52, 53) short and broad, with long dense setae. Surstylus (Figs 52, 53) with short stout setae on inner distal surface. Cerci (Figs 52, 53) relatively broad, shorter than surstylus, with short dense setae. Hypandrium (Figs 54, 55) broadly rounded anteriorly, strongly arched medially, with one pair of bifid, long anterior lobes. Phallapodeme (Figs 54, 55) extremely long, slightly curved. Pregonite (Fig. 54) long, narrow, band-like. Phallic plate (Fig. 55) strongly thickened, divided into two articulating sclerites. Distiphallus (Figs 54, 55) extremely short, nearly half as long as phallapodeme, lacking apical “glans”, membrane microtrichose. Ejaculatory apodeme (Fig. 55) small.

**Female.** Antennal first flagellomere lacking process (Fig. 46); clypeus thick, bulbous. Other characters same as those of male.

#### Etymology.

This species is named after its type locality.

#### Distribution.

**Figure 56.**
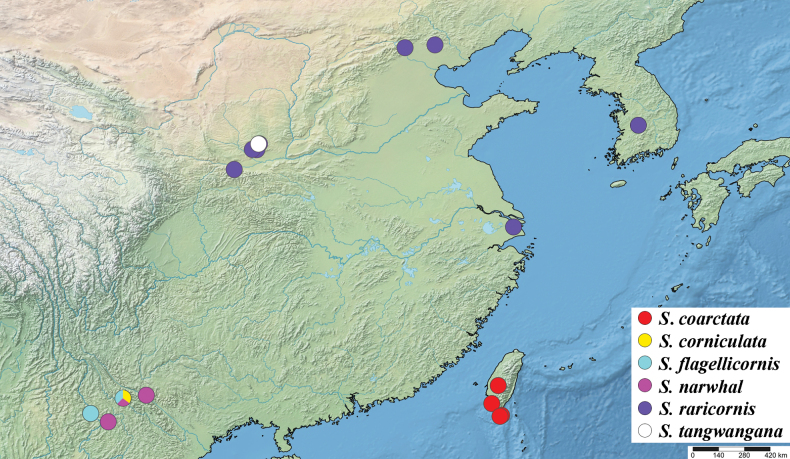
Known distribution of species of the *Strongylophthalmyiacoarctata* subgroup in China and South Korea.

China – Shaanxi: Tongchuan (Fig. 56).

#### Comparative notes.

This new species is similar to *S.corniculata* sp. nov., but can be distinguished from it in the following characters: frons black, at most anterior margin slightly paler (anterior half of frons yellowish brown in *S.corniculata* sp. nov.); first flagellomere of male with a small, bump-like process (with a small, conical, apically acute process in *S.corniculata* sp. nov.); thorax with two posterior supra-alar setae (one in *S.corniculata* sp. nov.); apical section of M_1+2_ straight (slightly arched in *S.corniculata* sp. nov.); distiphallus nearly half as long as phallapodeme (distinctly shorter than half of length of phallapodeme in *S.corniculata* sp. nov.).

This new species also resembles *S.gibbifera* Shatalkin, 1993, but differs in having a different color pattern on the frons, first flagellomere, and the mid and hind femora and hind tibia, and in the different shape of the antennal process.

## ﻿Discussion

The present study is part of our ongoing taxonomic study of the Chinese Strongylophthalmyiidae, documenting the *Strongylophthalmyiacoarctata* subgroup from China, including three known species (two newly recorded in China) and three new species. All species are keyed.

The main characters currently used to distinguish species in the *S.coarctata* subgroup include body color (including color patterns on antenna and wing), male antennal morphology, and the inner basal process on male hind femur. In this study, we newly described the male genitalia of most Chinese species, and found that the morphology of the external genitalia, phallapodeme, and distiphallus differed among species in this subgroup: the external genitalia are short and broad in some species, while in others they are long and narrow; the phallapodeme is straight or clearly curved, and the relative length of the distiphallus varies among species; the distiphallus of different species has different forms of tricha on the membrane and apical “glans”, and the apical “glans” is absent in *S.corniculata* sp. nov. and *S.tangwangana* sp. nov. These male genital characters are useful for species-level identification. In addition, the thoracic chaetotaxy varies between species, which may also be helpful in identifying female specimens that lack male-specific features.

The *S.coarctata* subgroup has high diversity in the Oriental Realm. However, most of the specimens currently available to us were collected from several scattered localities in China. There is no doubt that a considerable number of other species could be discovered in China (especially in southern China) following more thorough field investigations.

## Supplementary Material

XML Treatment for Strongylophthalmyia

XML Treatment for Strongylophthalmyia coarctata

XML Treatment for Strongylophthalmyia corniculata

XML Treatment for Strongylophthalmyia flagellicornis

XML Treatment for Strongylophthalmyia narwhal

XML Treatment for Strongylophthalmyia raricornis

XML Treatment for Strongylophthalmyia tangwangana

## ﻿Additional information

### Author ORCIDs

Jiale Zhou https://orcid.org/0000-0002-8901-3187

Neal L. Evenhuis https://orcid.org/0000-0002-1314-755X

Ding Yang https://orcid.org/0000-0002-7685-3478

### Data availability

All of the data that support the findings of this study are available in the main text or Supplementary Information.
